# Effects of Arthrocen, an avocado/soy unsaponifiables agent, on inflammatory mediators and gene expression in human chondrocytes

**DOI:** 10.1002/2211-5463.12176

**Published:** 2017-01-09

**Authors:** Ramin Goudarzi, Jared F. Taylor, Puya G. Yazdi, Brian A. Pedersen

**Affiliations:** ^1^ Pharmin USA, LLCSan JoseCAUSA; ^2^ Systomic HealthLos AngelesCAUSA; ^3^ Division of Rheumatology, Allergy and ImmunologyDepartment of MedicineUniversity of California, San DiegoLa JollaCAUSA

**Keywords:** Arthrocen, avocado soy unsaponifiables, chondrocytes, eiscosanoids, osteoarthritis, RNA‐Sequencing

## Abstract

Osteoarthritis (OA) is a chronic joint disease characterized by pain and stiffness. Recently, there has been great interest in the use of plant‐derived compounds and supplements in managing the symptoms of OA. Arthrocen is a plant‐based supplement consisting of avocado and soy unsaponifiable extracts in a 1 : 2 ratio. In an effort to unravel the potential mechanisms of its action on the cellular level, we utilized an *in vitro* assay to study its effects on cultured human chondrocytes. By pairing this assay with protein arrays on inflammatory markers, RNA‐Seq with downstream pathway analysis, and lipidomics on eicosanoids, we were able to further define its action at the molecular level. Specifically, we found a role for Arthrocen in attenuating the inflammatory response both at the protein and mRNA level. Furthermore, we discovered that Arthrocen diminished prostaglandin E2 (PGE2) levels in response to an inflammatory trigger. Additionally, unlike traditional COX‐2 inhibitors, this response rather specifically attenuated PGE2 levels in the presence of inflammation and without lowering levels of other eicosanoids. This implies that Arthrocen could potentially bring about the reduced pain produced by COX‐2 inhibitors without the known side effects of COX‐2 inhibition.

**Abbreviations**
12,13‐DiHOME: (±)12,13‐dihydroxy‐9Z‐octadecenoic acid
ASU: avocado/soy unsaponifiable
GO: gene ontology
NSAIDs: nonsteroidal anti‐inflammatory drugs
OA: osteoarthritis
PGE2: prostaglandin E2
RNA‐Seq: RNA‐Sequencing

Osteoarthritis (OA) is a chronic joint disease whose main symptoms are pain and stiffness. OA is thought to be a result in part caused by mechanical stress and this is consistent with the observation that it most commonly affects the weight‐bearing joints in the knees and hips. The disease is characterized by its progressive nature, specifically, damage of articular cartilage, bone remodeling, new bone formation, synovial inflammation, and fibrosis 1. Breakdown of the articular cartilage with underlying remodeling of the bone is the hallmark of OA. Cartilage is avascular and aneural and contains just one cell type, the chondrocyte. About 95% of articular cartilage volume is extracellular matrix, predominantly composed of type II collagen and the proteoglycan, aggrecan 2. Chondrocytes are responsible for maintaining homeostatic cartilage turnover to renew and respond to changes in the mechanical environment, but excessive matrix catabolism can be driven by excessive mechanical joint loading, cytokines, growth factors, and fragments of the extracellular matrix 3. The significance of joint inflammation in contributing to tissue breakdown is unclear. However, infiltration of mononuclear cells into the synovial membrane is observed in human OA and there is much evidence to support activation of the innate immune system in disease 4, 5. It seems likely that inflammation, when present, can exacerbate tissue breakdown and contribute to painful episodes of disease. Patients with OA suffer from decreased joint function, localized inflammation, and pain symptoms. Current therapies are mainly palliative in nature and aim to slow disease progression or provide analgesia before joint replacement is ultimately indicated.

There has been increasing interest in the use of botanical extracts as therapeutics for OA. Specifically, the biologically active class of lipids called unsaponifiable lipids from oil extracts of avocados and soybeans [avocado/soy unsaponifiables (ASU)]. Phytosterols are the major component of ASU with beta‐sitosterol, campesterol, and stigmasterol predominating. The hypocholesterolemic effect of phytosterols is well characterized and likely mediated through their ability to inhibit cholesterol absorption and disrupt endogenous cholesterol biosynthesis 6. More recently, studies have demonstrated anti‐ inflammatory properties, antioxidant and analgesic properties of phytosterols 7, 8, 9, 10. Clinical work has teased the possibility of ASU having an analgesic and disease‐modifying effect in individuals with OA.

Arthrocen is an ASU‐containing supplement. Arthrocen's avocado and soy unsaponifiables are extracted and sold in bulk form under the trade name AvoVida. It contains avocado and soy unsaponifiable extracts in a 1 : 2, respectively. As per mass spectroscopy, it is composed primarily of dihydrocholesterol, campesterol, stigmastanol, and β‐sitosterol and its sterol composition is better defined in comparison to other formulations of avocado and soy unsaponifiable extracts 11. Arthrocen is manufactured within US Food and Drug Administration (FDA)‐inspected facilities under current Good Manufacturing Practices (cGMP). Arthrocen is classified as a human prescription drug in some countries while in others like the United States it is characterized as a dietary supplement. In order to determine the possible mechanism of its action in the pathophysiology of OA, we utilized human chondrocytes in an *in vitro* system. Chondrocytes are the only cell type found in healthy cartilage and serve to produce and maintain the cartilaginous matrix. This matrix is primarily composed of proteoglycans and cartilage 2. Specifically, we set out to test Arthrocen's effects at therapeutic equivalent doses on gene transcription, select immune protein expression levels, and eicosanoids in human chondrocytes.

## Materials and methods

The experiment consisted of giving a therapeutic equivalent dose of Arthrocen or control media to each cell type in triplicate. The therapeutic equivalent dose was calculated as 25 μg·mL^−1^ of Arthrocen based on previous literature 7. Arthrocen is manufactured as per a patented process (U.S. patent EP2464248A2). The source of the ASU in the final product is sold as AvoVida commercially and is the same source as that used to make the ASU product that was utilized in the referenced study. Hence, in order to make a direct comparison with previously published peer‐reviewed literature, we used the same concentration of ASU as that in the referenced study. Specifically, 25 μg·mL^−1^ of Arthrocen was added to cell media supplemented with 10% FBS. Additionally, we also induced a proinflammatory response with the addition of LPS. Hence, in total, we had four groups: first, chondrocytes with vehicle; second, chondrocytes with Arthrocen; third, chondrocytes with LPS stimulation; fourth, chondrocytes with Arthrocen and LPS stimulation. In all situations, control refers to the media supplemented with FBS that does not include the Arthrocen. At the conclusion of the cell culture part of the study, cells and their corresponding culture supernatants were harvested.

### Chondrocyte culture and avocado soy unsaponifiable preparation

Primary human chondrocytes isolated from normal human articular cartilage were purchased from PromoCell (Heidelberg, Germany) and cultured in PromoCell Chondrocyte Growth Medium (Promocell) as per the supplier's protocol with 100 U·mL^−1^ penicillin and 100 μg·mL^−1^ streptomycin (Life Technologies, Carlsbad, CA, USA). All experiments were performed at passage 5 and the chondrocytes were obtained from normal human articular cartilage from the knee. Arthrocen (Pharmin USA, LLC, San Jose, CA, USA), a compound of avocado : soy unsaponifiables in a 1 : 2 ratio, as per dry weight was dissolved in 100% ethanol at 50 °C for 60 min with continuous mixing. Human chondrocytes were plated in tissue culture‐treated six‐well plates (Falcon, Tewksbury, MA, USA) at a density of 5 × 10^5^ cells per well and incubated at 37 °C, 5% CO_2_ for 72 h with either vehicle (ethanol) or soy : avocado unsaponifiables (25 μg·mL^−1^). After pretreatment, the human chondrocytes were activated with LPS (Sigma‐Aldrich, St. Louis, MO, USA) at a concentration of 20 ng·mL^−1^ for 6 h. Culture supernatants were then collected and snap frozen in liquid nitrogen. About 1 mL of ice‐cold PBS was then added to the wells and the chondrocytes were harvested using a cell scraper. Cell pellets were then collected by centrifugation at 400 ***g*** for 3 min at 4 °C. The overlying PBS was then aspirated and the cell pellets were snap frozen in liquid nitrogen.

### Analysis of inflammatory factors in culture supernatants

Frozen culture supernatants were supplied in triplicate to RayBiotech (Norcross, GA, USA) for the determination of protein concentrations using their Quantibody^®^ Human Inflammation Array 3 Kit (RayBiotech). This kit determines the concentration of the following 40 cytokines/chemokines: BLC (CXCL13), Eotaxin‐1 (CCL11), Eotaxin‐2 (MPIF‐2/CCL24), GCSF, GM‐CSF, I‐309 (TCA‐3/CCL1), ICAM‐1 (CD54), IFN‐gamma, IL‐1 alpha (IL‐1 F1), IL‐1 beta (IL‐1 F2), IL‐1 ra (IL‐1 F3), IL‐2, IL‐4, IL‐5, IL‐6, IL‐6 R, IL‐7, IL‐8 (CXCL8), IL‐10, IL‐11, IL‐12 p40, IL‐12 p70, IL‐13, IL‐15, IL‐ 16, IL‐17A, MCP‐1 (CCL2), M‐CSF, MIG (CXCL9), MIP‐1 alpha (CCL3), MIP‐1 beta (CCL4), MIP‐1 delta (CCL15), PDGF‐BB, RANTES (CCL5), TIMP‐1, TIMP‐2, TNF alpha, TNF beta (TNFSF1B), TNF RI (TNFRSF1A), TNF RII (TNFRSF1B).

### Eicosanoid analysis

Snap frozen cell pellets were supplied in triplicate to the LIPID MAPS^®^ Lipidomics Core at the University of California, San Diego for a comprehensive eicosanoid panel analysis. Eicosanoids were prepared and analyzed as described previously 12.

### RNA extraction and RNA‐sequencing

Total RNA from cell pellets in triplicate per group was isolated with Trizol (Life Technologies, Carlsbad, CA, USA) as per the manufacturer's instructions. These pellets consisted of the cells from which the supernatants were collected and used to quantify the cytokines/chemokines. The concentration of the isolated RNA was quantified using Nanodrop (Thermo Scientific, Waltham, MA, USA). Total RNA was then supplied to Applied Biological Materials, Inc. (Richmond, BC, Canada) for downstream processing. Samples underwent poly(A) enrichment prior to RNA‐sequencing (~ 8 million reads, 1 × 75 bp single end). The resulting FASTQ files for each replicate were then processed with tophat (version 2.1.0) to align the reads to the reference human genome (GRCh38/hg38). The mapped reads were assembled using cufflinks (version 2.2.1) with default parameters and as per ENCODE (Encyclopedia of DNA Elements) Consortium guidelines to determine transcript expression 13, 14. Merged transcriptome assemblies were then processed with the cuffdiff tool within Cufflinks to identify differentially expressed genes. See Table S2 [Differentially expressed mRNA transcripts as per cuffdiff analyses of RNA‐Sequencing (RNA‐Seq) data] for differentially expressed gene transcripts between experimental groups.

### Statistical analysis

Statistical significance between groups for the protein array and the eicosanoid panel data was evaluated using one‐way ANOVA with *P*‐values calculated as per *post‐hoc* Tukey test. Differences were deemed statistically significant at *P* < 0.05. The Cufflinks package with default setting was utilized to identify differentially expressed gene transcripts (cit as per below). Gene ontology (GO) clustering for biological processes was performed using david 6.7 15. GOrilla was utilized to visualize enriched GO terms for biological processes with a *P*‐value threshold of < 10^−9^ 16.

## Results

**Figure 1:**
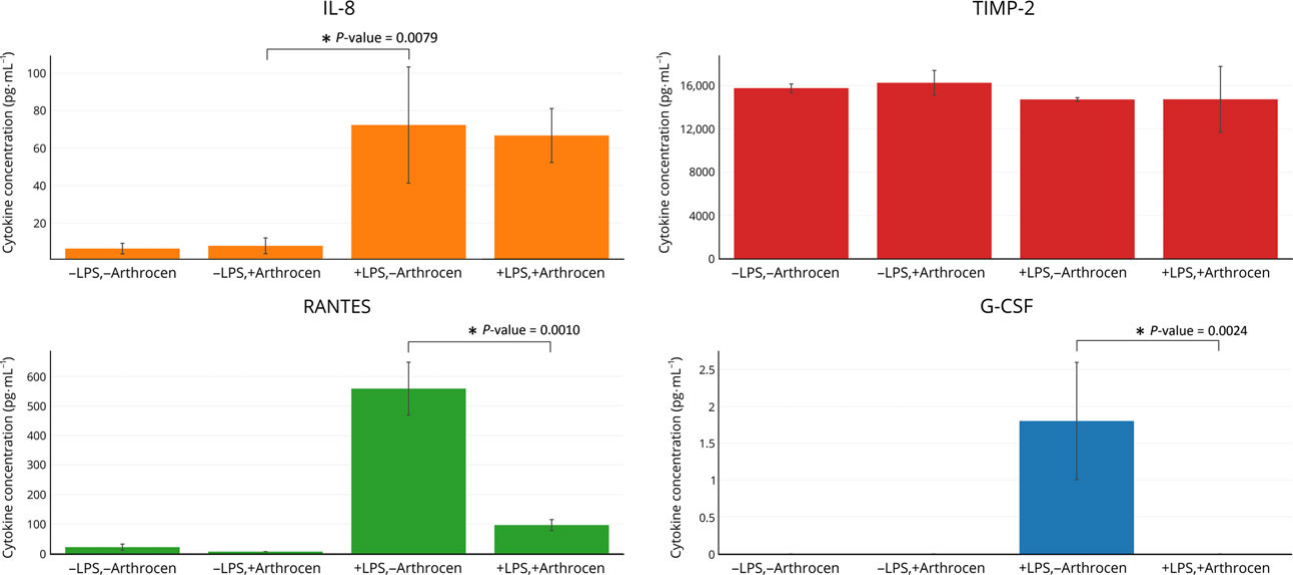
Effects of Arthrocen on proinflammatory cytokines as per ELISA. The results for IL‐8, TIMP‐2, RANTES, and G‐CSF are shown. Overall effect for a given cytokine is noted by a * between selected groups and representative of a *P*‐value < 0.05 as per one‐way ANOVA. Actual *P*‐values are as displayed on the panels.

In an effort to examine the effects of Arthrocen on inflammatory cytokines we screened the secreted a protein profile of the human chondrocytes using a protein array assay. Chondrocytes were either incubated in media alone or in media with Arthrocen for 72 h and then for an additional 6 h with either vehicle alone or LPS. Of the 40 proteins analyzed, only 13 were detectable (as defined by the presence in all replicates of triplicate testing for an experimental group; Table S1). Of these 13 proteins, only three demonstrated a statistically significant difference upon comparison between groups. As expected, LPS stimulation increased the levels of IL‐8, RANTES, and G‐CSF. Interestingly, preincubation with Arthrocen attenuated the secretion of RANTES and G‐CSF, but not IL‐8 (Fig. 1). This result is indicative of a modulatory effect of Arthrocen on the proinflammatory changes induced by LPS.

**Figure 2:**
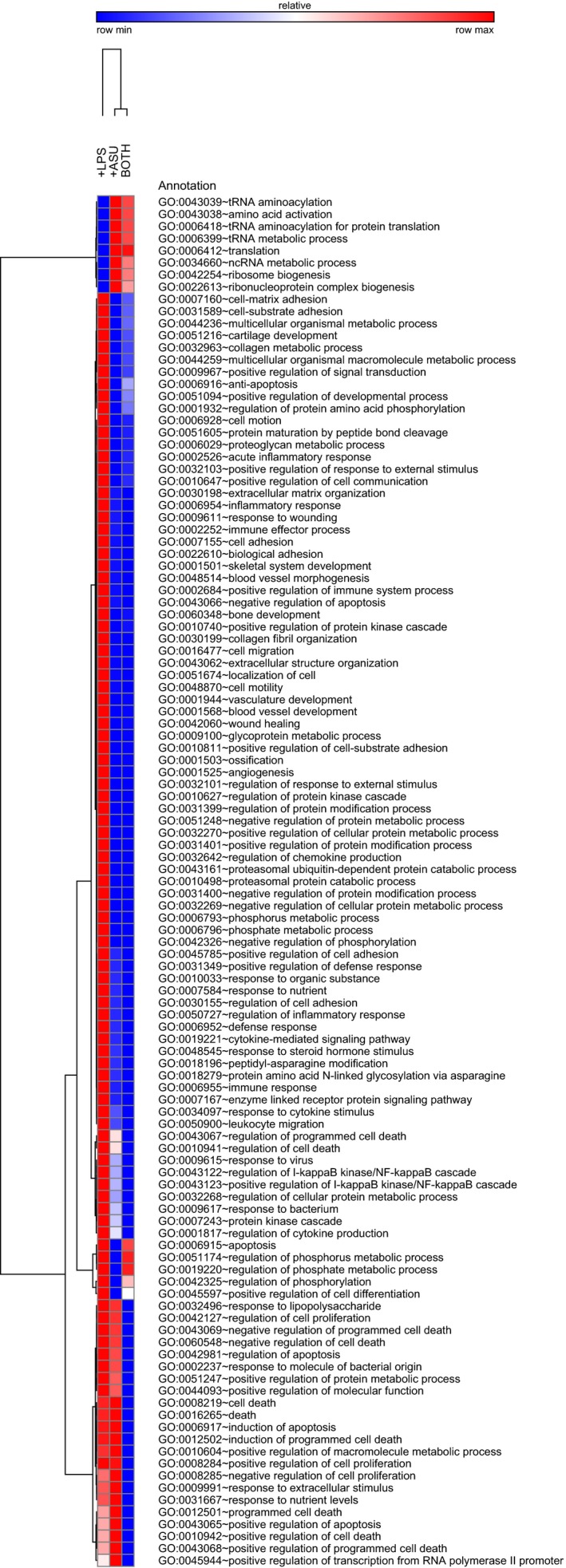
Graphical display of the effects of Arthrocen on gene expression as per clustering analyses of RNA‐Sequencing data. Hierarchical clustering of GO clusters for biological processes with a statistically significant difference (*q*‐value < 0.05) in a least one of the indicated comparisons.

Encouraged by the results of the protein array, we proceeded to investigate the effects of Arthrocen on global gene expression by performing RNA‐Seq of poly(A)‐enriched RNA from the same samples used in the protein array. Gene ontology (GO) analysis on the RNA‐Seq data was then utilized to highlight the biological processes that are potentially affected by Arthrocen. As can be seen by the hierarchical clustering of the GO terms (Fig. 2), the preincubation with Arthrocen is associated with a gene expression profile that is relatively stable upon acute stimulation with LPS. Additionally, the clustering data suggest that Arthrocen reduces gene expression of groups of genes involved in functions characteristic of chondrocytes.

**Figure 3:**
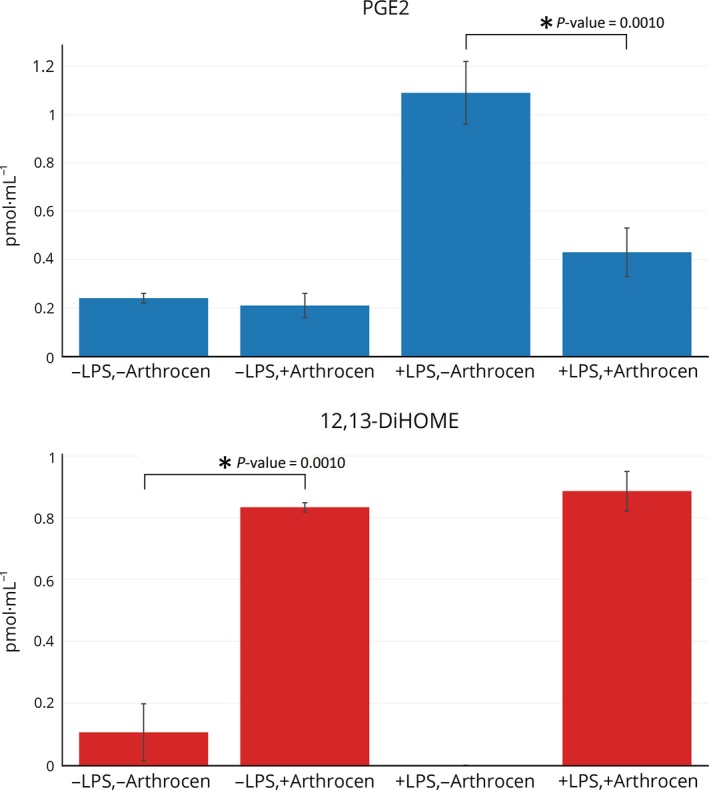
Effects of Arthrocen on eicosanoids. The results for PGES and 12,13‐DiHOMEIare shown. Overall effect for a given eicosanoid is noted by a * between selected groups and representative of a *P*‐value < 0.05 as per one‐way ANOVA.

As some studies have indicated that the ingestion of avocado soy unsaponifiables decreases the usage of nonsteroidal anti‐inflammatory drugs (NSAIDs), we sought to determine whether Arthrocen had an effect on eicosanoid levels 17, 18. An eicosanoid panel was performed, as these molecules can serve as messengers of the pain pathway and NSAIDs inhibit the synthesis of some eicosanoids. Samples were analyzed for 132 different eicosanoids. The presence of 18 different eicosanoids was detected (as defined by the presence in all replicates of triplicate testing for an experimental group; Table S3). However, a significant difference between treatment groups was detected in only two different eicosanoids [prostaglandin E2 (PGE2) and (±)12,13‐dihydroxy‐9Z‐octadecenoic acid (12,13‐DiHOME)]. As shown in Fig. 3, PGE2 levels were increased upon LPS stimulation and attenuated with Arthrocen. 12,13‐DiHOME was present in both experimental groups that were exposed to Arthrocen and implies that Arthrocen stimulates its production.

## Discussion

Analyses were performed to determine the effects of ASU on chondrocytes as this cell type is thought to play a primary role in the pathogenesis of osteoarthritis. These studies profiled ASU's influence on the basal state, as well as, its potential role to alter the response to an inflammatory trigger in chondrocytes. This was investigated by quantifying known markers of inflammation at the protein level, assessing for changes in gene expression using next‐generation sequencing technology, and profiling known mediators (eicosanoids) of cell signaling that play a role in pain and inflammation. This unbiased approach was employed to better understand the potential role of Arthrocen in influencing an immune response at a global level and then be able to elucidate the specific molecules it is influencing.

As demonstrated by Fig. 1, Arthrocen had a statistically significant effect on reducing G‐CSF and RANTES in the presence of the proinflammatory agent LPS. G‐CSF stimulates the production and survival of both granulocytes and neutrophils 19. RANTES is a chemokine for T cells, eosinophils, and basophils, which plays a role in recruiting leukocytes into inflammatory sites 20, 21, 22. These findings are consistent with a role of ASU in diminishing the progression of OA by directly influencing the production of known inflammatory agents at the level of the chondrocyte.

Next, we performed RNA‐seq to quantify changes in messenger RNA levels that are affected by Arthrocen. As shown in Fig. 2, Arthrocen exposure resulted in widespread and statistically significant changes to numerous transcripts involved in diverse pathways linked to inflammation. Most importantly, the addition of Arthrocen was able to attenuate the effects of the proinflammatory agent LPS. As stated earlier, this implies that Arthrocen, at least in part, works at the level of gene transcription in chondrocytes to attenuate inflammation in OA. Gene ontology enrichment analysis, as per genes differentially expressed between LPS‐stimulated cells and LPS‐stimulated cells preincubated with Arthrocen, recapitulated the relationship between enriched terms (Fig. S1). This analysis further highlights that many of the affected pathways are involved in the response to a nutrient or steroid, synthesis of extracellular matrix, apoptosis, and chemotaxis. Involvement of these processes further supports the biological plausibility of ASU influencing the pathogenesis of osteoarthritis at the level of the chondrocyte.

We then performed a lipidomic analysis to study the effects of Arthrocen on eiconasoids. Eicosanoids are signaling molecules that are derived from the oxidation of 20‐carbon fatty acids. They are thought to induce changes over many cellular and physiological systems: cellular growth, inflammation, and immunity after the intake of toxic compounds or exposures 23. Hence, some are considered local hormones. Our analysis demonstrated that in chondrocytes, Arthrocen was able to rather specifically attenuate the effects of LPS on PGE2 production. PGE2 is a potent signaling molecule with diverse effects that includes numerous roles in inflammation and pain signaling.^24^ PGE2 is implicated in bone remodeling as increased levels lead to bone resorption by osteoclasts 25. Prostaglandin E synthase is the enzyme that converts PGH2 to PGE2. There are three different isoforms of prostaglandin E synthase. One isoform [prostaglandin E synthase 2 (PTGES2)] was differentially regulated in the comparison between the two LPS‐stimulated groups that differed only in Arthrocen addition with expression levels 2.1 times greater in the Arthrocen addition group (q‐value = 0.001). Given the decreased levels of PGE2 in the LPS with Arthrocen group, this implies that perhaps a post‐translational modification of PTGES2 is regulated by Arthrocen and plays a role in diminishing the levels of PGE2. Of note, 12,13‐DiHOME was present in both experimental groups that were exposed to Arthrocen. This suggests that Arthrocen stimulates its production or contains a metabolyte required for its synthesis. 12,13‐DiHOME is synthesized from 12,13‐EpOME by soluble epoxide hydrolase (EPHX2). Gene expression of EPHX2, as per our own RNA‐Seq data, was unchanged upon exposure to Arthrocen. Interestingly, 12,13‐EpOME is synthesized from linoleic acid (an essential fatty acids for humans) and linoleic acid is the most abundant polyunsaturated fatty acid of both avocados and soybeans. 12,13‐DiHOME has previously been shown to have neutrophil chemotactic activity but inhibits neutrophil respiratory burst activity 26. While it is true that osteoarthritic joints exhibit lower inflammatory cellular infiltrates than other forms of arthritis, studies have shown that low levels of infiltrates are found and appear to play a role in OA. Additionally, increased 12,13‐DiHOME concentrations in the plasma seem to inhibit or depress the immune response 27. Hence, Arthrocen's ability to increase 12,13‐DiHOME in chondrocytes could exert an immune depressing effect either through a general increase in its level on a system‐wide basis or a direct immunosuppressive effect whenever neutrophils happen to infiltrate the OA joint space.

Overall, the underlying theme of this three‐pronged approach to better understand the effects of Arthrocen is that it had a minimal effect on the basal status of the chondrocytes. However, Arthrocen was able to greatly attenuate the response to a strong proinflammatory trigger, LPS in our model. This was evident at the level of gene transcription (mRNA), proteins involved in immune responses, and signaling messengers (eicosanoids) of pain and inflammation. When individual mediators that are significantly altered are studied in fine detail, it is apparent that Arthrocen can dampen the response of known mediators of inflammation and pathological processes. Many examples of this are very well described mediators of such processes in the scientific and medical literature (RANTES, TNF receptors, and prostaglandin E2). One of the more striking examples of this was the relative specificity of Arthrocen on a comprehensive analysis of eicosanoid levels produced by the chondrocytes. Arthrocen exposure on the chondrocytes significantly diminished prostaglandin E2 levels in response to an inflammatory trigger. Interestingly, prostaglandin E2 is an active metabolite involved in pain signaling that is downregulated by multiple medications used to treat pain symptoms (COX‐2 inhibitors such as celecoxib, rofecoxib, etoricoxib) of osteoarthritis and perhaps provides a mechanism by which joint pain is reduced. Additionally, unlike traditional COX‐2 inhibitors, ASU appears to only bring prostaglandin E2 levels back to normal in the presence of inflammation without significantly lowering the levels of other eicosanoids 28, 29. This implies that ASU could potentially bring about the reduced pain produced by COX‐2 inhibitors without the known side effects of COX‐2 inhibition. Nonetheless, one of the limitations of this study was that it was conducted in a single cell line in triplicate only. This was done, in order to keep the inherent variability of primary cell lines to a minimum. Controlling for this variability facilitates the elucidation of the various cellular mechanisms potentially involved. In essence, our work aimed to be a descriptive study of the biological effects of Arthrocen. Additional work with more primary cells, and/or further animal and clinical studies, are needed before drawing bigger conclusions from the data.

In conclusion, our *in vitro* datasets on the effects of Arthrocen on chondrocytes as an assay for OA pathogenesis has demonstrated a possible and or potential role for Arthrocen in attenuating the inflammatory response at the cellular level that needs further scientific study. Next steps should entail clinical studies that involve testing the clinical efficacy of Arthrocen in reducing pain symptoms and radiographic progression in OA, and further animal and cell culture work that would help more fully elucidate and or confirm our findings.

## Conflicts of interest

Ramin Goudarzi owns equity in Pharmin USA which sells and distributes Arthrocen. Puya Yazdi owns equity in Systomic Health.

## Author contributions

RG conceived the project. RG, JT, PGY, and BAP contributed to the design of the project. JT, PGY, and BAP acquired the data, analyzed/interpreted the data, and wrote the manuscript.

## Supporting information


**Fig. S1.** Graphical representation of enriched GO terms for biological processes for the comparison of chondrocytes stimulated in the absence or presence of Arthrocen.

**Table S1.** Protein concentrations of all 40 cytokines/chemokines analyzed by protein array.

**Table S2.** Differentially expressed mRNA transcripts as per cuffdiff analyses of RNA‐Sequencing data.

**Table S3.** Eicosanoid concentrations of all 132 eicosanoids analyzed.
