# Corrigendum to: Effects of Arthrocen, an avocado/soy unsaponifiables agent, on inflammatory mediators and gene expression in human chondrocytes

**DOI:** 10.1002/2211-5463.12481

**Published:** 2018-07-09

Within the Materials and Methods section of the paper by Goudarzi *et al*. 1, it was stated that Arthrocen is manufactured as per a patented process (U.S. patent EP2464248A2). However, this patent covers a general process for a ‘composition including an unsaponifiable fraction’ and does not relate to the manufacture of Arthrocen or the avocado and soya unsaponifiables it contains. Rather, Arthrocen possesses a US Trademark granted by the United States Patent and Trademark Office (USPTO 4397178).
