# Supplementary figures and images for: Effects of Arthrocen, an avocado/soy unsaponifiables agent, on inflammatory mediators and gene expression in human chondrocytes
Source: FEBS Open Bio. 2017 Jan 9;7(2):187–94. doi: 10.1002/2211-5463.12176 (PMC5292663; doi:10.1002/2211-5463.12176)

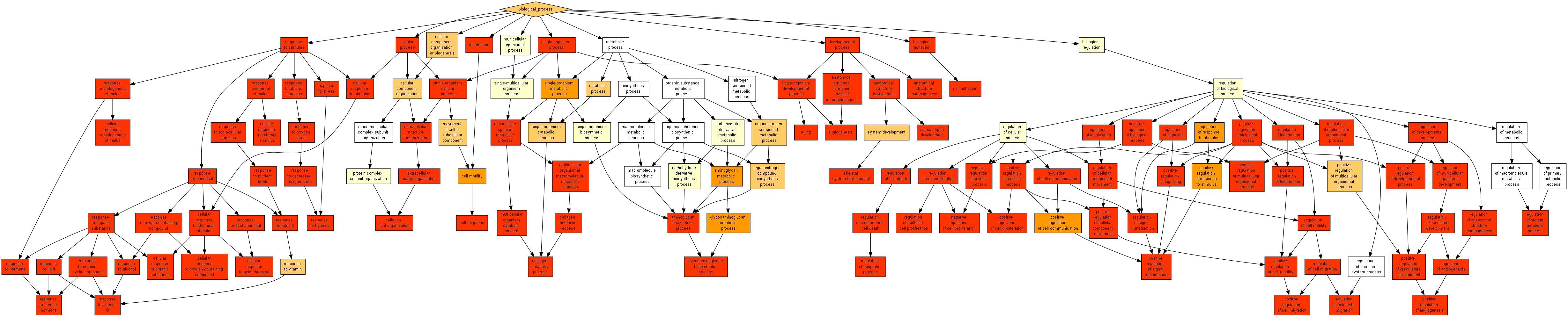

Supplement: Supplementary file 1 — Fig. S1. Graphical representation of enriched GO terms for biological processes for the comparison of chondrocytes stimulated in the absence or presence of Arthrocen. [file FEB4-7-187-s001.png]
